# Bis(diethyl­enetriamine)cadmium(II) diiodide

**DOI:** 10.1107/S1600536808008040

**Published:** 2008-05-03

**Authors:** Bei Huang, Zhigang Liu, Li Wang

**Affiliations:** a College of Life Sciences, Shenzhen University, Shenzhen 518060, People’s Republic of China; b Key Laboratory of Pesticides and Chemical Biology, Department of Chemistry, Central China Normal University, Wuhan, Hubei 430079, People’s Republic of China

## Abstract

In the title compound, [Cd(dien)_2_]I_2_, where dien = diethyl­enetriamine (C_4_H_13_N_3_), the Cd^II^ ion is in a distorted octa­hedral coordination environment. In the crystal structure, inter­molecular N—H⋯I hydrogen bonds link cations and anions into a three-dimensional network.

## Related literature

For related literature, see: Hynes *et al.* (1996 ▶); Biagini & Cannas (1970 ▶); Xiang *et al.* (2006 ▶); Hines *et al.* (2006 ▶).
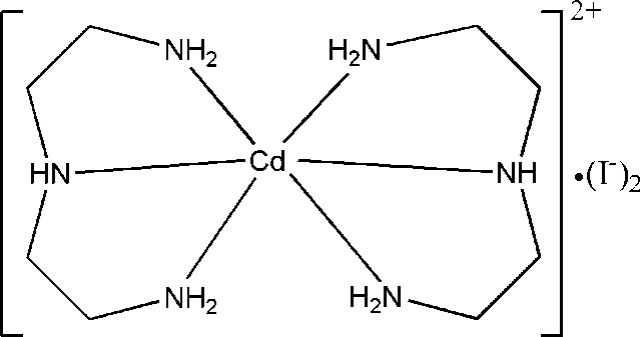


## Experimental

### 

#### Crystal data

[Cd(C_4_H_13_N_3_)_2_]I_2_
*M* *_r_* = 572.55
Monoclinic, 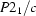

*a* = 9.8842 (9) Å
*b* = 15.1947 (11) Å
*c* = 12.4209 (9) Å
β = 100.204 (6)°
*V* = 1836.0 (3) Å^3^
*Z* = 4
Mo *K*α radiation
μ = 4.55 mm^−1^
*T* = 292 (2) K
0.30 × 0.30 × 0.20 mm

#### Data collection

Bruker SMART APEX CCD diffractometer
Absorption correction: multi-scan (*SADABS*; Sheldrick, 1996 ▶) *T*_min_ = 0.102, *T*_max_ = 0.177 (expected range = 0.232–0.403)
10779 measured reflections
3991 independent reflections
3214 reflections with *I* > 2σ(*I*)
*R*_int_ = 0.021

#### Refinement

*R*[*F* ^2^ > 2σ(*F* ^2^)] = 0.024
*wR*(*F* ^2^) = 0.057
*S* = 1.08
3991 reflections
154 parameters
H-atom parameters constrained
Δρ_max_ = 0.73 e Å^−3^
Δρ_min_ = −0.64 e Å^−3^

### 

Data collection: *SMART* (Bruker, 2001 ▶); cell refinement: *SAINT-Plus* (Bruker, 2001 ▶); data reduction: *SAINT-Plus*; program(s) used to solve structure: *SHELXS97* (Sheldrick, 2008 ▶); program(s) used to refine structure: *SHELXL97* (Sheldrick, 2008 ▶); molecular graphics: *PLATON* (Spek, 2003 ▶); software used to prepare material for publication: *PLATON*.

## Supplementary Material

Crystal structure: contains datablocks global, I. DOI: 10.1107/S1600536808008040/lh2585sup1.cif

Structure factors: contains datablocks I. DOI: 10.1107/S1600536808008040/lh2585Isup2.hkl

Additional supplementary materials:  crystallographic information; 3D view; checkCIF report

## Figures and Tables

**Table 1:** Selected bond lengths (Å)

Cd1—N5	2.352 (3)
Cd1—N1	2.357 (3)
Cd1—N2	2.365 (3)
Cd1—N3	2.366 (3)
Cd1—N6	2.380 (3)
Cd1—N4	2.381 (3)

**Table 2:** Hydrogen-bond geometry (Å, °)

*D*—H⋯*A*	*D*—H	H⋯*A*	*D*⋯*A*	*D*—H⋯*A*
N3—H3*C*⋯I2^i^	0.90	2.77	3.673 (3)	176
N6—H6*C*⋯I2^i^	0.90	2.82	3.709 (3)	168
N3—H3*D*⋯I1^ii^	0.90	2.87	3.759 (3)	168
N4—H4*D*⋯I1^ii^	0.90	3.02	3.873 (3)	159
N5—H5⋯I1^iii^	0.91	2.87	3.778 (3)	174
N1—H1*D*⋯I2	0.90	2.85	3.685 (3)	155
N2—H2⋯I1	0.91	2.98	3.869 (3)	167
N4—H4*C*⋯I1	0.90	2.96	3.789 (3)	153
N6—H6*D*⋯I2	0.90	2.86	3.751 (3)	169

Symmetry codes: (i) 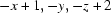; (ii) 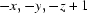; (iii) 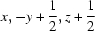.

**Fig. 1.**
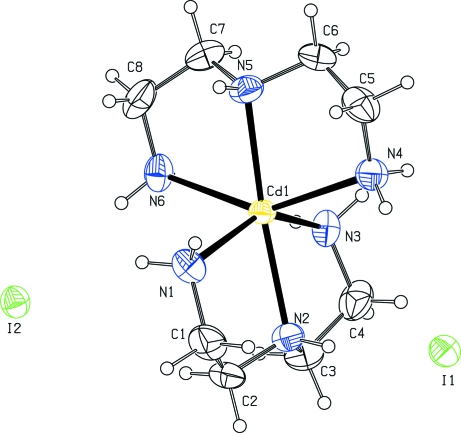
The molecular structure, showing 50% probability displacement ellipsoids and H atoms as small spheres.

**Fig. 2.**
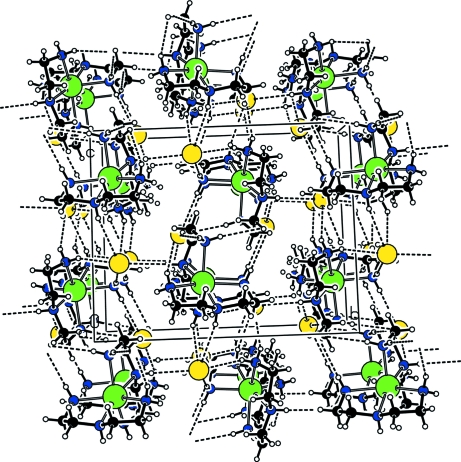
Part of the crystal structure showing hydrogen bonds as dashed lines.

## supplementary crystallographic information

### Comment

The goal of our research has been to determine the capability of a number of linear multidentate ligands to induce extended structures in cadmium compounds. Previously, some ligands containing diethylenetriamine and their metal coordination compounds have been studied (Hines *et al.*,2006; Biagini & Cannas, 1970; Hynes, *et al.*, 1996; Xiang, *et al.*, 2006).

In the molecular structure, the Cd^II^ ion is coordinated by six N atoms from two diethylene triamine ligands, forming a distorted octahedral coordination geometry (Fig. 1). In the crystal structure, intermolecular N–H···I hydrogen bonds link the cations and anions into a three-dimensional network (Fig.2).

### Experimental

Diethylenetriamine (0.21 g, 2.0 mmol) in 10 ml water was added slowly to a CdAc_2_.2H_2_O (0.27 g, 1.0 mmol) solution in 10 ml water and KI (0.33 g, 2.0 mmol) solution in 10 ml water. The mixture was stirred for 1 h. After filtration, the colourless solution was allowed to stand at room temperature. Colourless block-shaped crystals suitable for X-ray analysis were obtained in several days in 50% yield.

### Refinement

H atoms were placed in calculated positions with C—H = 0.97 Å, N—H = 0.90Å (NH_2_) N—H = 0.91Å (NH) and *U*_iso_=1.2*U*_eq_(C,*N*).

### Crystal data

**Table:**

[Cd(C_4_H_13_N_3_)_2_]I_2_	*F*_000_ = 1080
*M**_r_* = 572.55	*D*_x_ = 2.071 Mg m^−^^3^
Monoclinic, *P*2_1_/*c*	Mo *K*α radiation λ = 0.71073 Å
Hall symbol: -P 2ybc	Cell parameters from 1123 reflections
*a* = 9.8842 (9) Å	θ = 2.4–26.8º
*b* = 15.1947 (11) Å	µ = 4.55 mm^−^^1^
*c* = 12.4209 (9) Å	*T* = 292 (2) K
β = 100.204 (6)º	Block, colorless
*V* = 1836.0 (3) Å^3^	0.30 × 0.30 × 0.20 mm
*Z* = 4	

### Data collection

**Table:**

Bruker SMART APEX CCD diffractometer	3991 independent reflections
Radiation source: fine focus sealed Siemens Mo tube	3214 reflections with *I* > 2σ(*I*)
Monochromator: graphite	*R*_int_ = 0.021
*T* = 292(2) K	θ_max_ = 27.0º
0.3° wide ω exposures scans	θ_min_ = 2.1º
Absorption correction: multi-scan(SADABS; Sheldrick, 1996)	*h* = −12→6
*T*_min_ = 0.102, *T*_max_ = 0.177	*k* = −19→19
10779 measured reflections	*l* = −15→15

### Refinement

**Table:**

Refinement on *F*^2^	Secondary atom site location: difference Fourier map
Least-squares matrix: full	Hydrogen site location: inferred from neighbouring sites
*R*[*F*^2^ > 2σ(*F*^2^)] = 0.024	H-atom parameters constrained
*wR*(*F*^2^) = 0.057	*w* = 1/[σ^2^(*F*_o_^2^) + (0.0279*P*)^2^] where *P* = (*F*_o_^2^ + 2*F*_c_^2^)/3
*S* = 1.08	(Δ/σ)_max_ = 0.001
3991 reflections	Δρ_max_ = 0.73 e Å^−^^3^
154 parameters	Δρ_min_ = −0.64 e Å^−^^3^
Primary atom site location: structure-invariant direct methods	Extinction correction: none

### Special details

**Table:**

Geometry. All e.s.d.'s (except the e.s.d. in the dihedral angle between two l.s. planes) are estimated using the full covariance matrix. The cell e.s.d.'s are taken into account individually in the estimation of e.s.d.'s in distances, angles and torsion angles; correlations between e.s.d.'s in cell parameters are only used when they are defined by crystal symmetry. An approximate (isotropic) treatment of cell e.s.d.'s is used for estimating e.s.d.'s involving l.s. planes.
Refinement. Refinement of *F*^2^ against ALL reflections. The weighted *R*-factor *wR* and goodness of fit *S* are based on *F*^2^, conventional *R*-factors *R* are based on *F*, with *F* set to zero for negative *F*^2^. The threshold expression of *F*^2^ > σ(*F*^2^) is used only for calculating *R*-factors(gt) *etc*. and is not relevant to the choice of reflections for refinement. *R*-factors based on *F*^2^ are statistically about twice as large as those based on *F*, and *R*- factors based on ALL data will be even larger.

### Fractional atomic coordinates and isotropic or equivalent isotropic displacement parameters (Å^2^)

**Table:**

	*x*	*y*	*z*	*U*_iso_*/*U*_eq_	
Cd1	0.22542 (2)	0.082844 (14)	0.762034 (17)	0.04124 (7)	
C1	0.4649 (5)	0.1808 (3)	0.6705 (4)	0.0826 (13)	
H1A	0.5485	0.2154	0.6864	0.099*	
H1B	0.4167	0.1972	0.5984	0.099*	
C2	0.5008 (4)	0.0855 (3)	0.6713 (4)	0.0794 (13)	
H2A	0.5577	0.0742	0.6167	0.095*	
H2B	0.5530	0.0697	0.7423	0.095*	
C3	0.3956 (5)	−0.0636 (3)	0.6679 (4)	0.0875 (14)	
H3A	0.4529	−0.0734	0.7388	0.105*	
H3B	0.4414	−0.0892	0.6124	0.105*	
C4	0.2589 (5)	−0.1067 (3)	0.6646 (4)	0.0842 (14)	
H4A	0.2022	−0.0975	0.5933	0.101*	
H4B	0.2716	−0.1696	0.6756	0.101*	
C5	−0.0645 (4)	0.1671 (3)	0.6612 (4)	0.0790 (12)	
H5A	−0.1550	0.1624	0.6162	0.095*	
H5B	−0.0314	0.2267	0.6550	0.095*	
C6	−0.0731 (4)	0.1480 (3)	0.7764 (4)	0.0792 (12)	
H6A	−0.1367	0.1887	0.8012	0.095*	
H6B	−0.1080	0.0888	0.7820	0.095*	
C7	0.0734 (5)	0.1198 (4)	0.9566 (3)	0.0876 (14)	
H7A	0.0390	0.0598	0.9525	0.105*	
H7B	0.0179	0.1544	0.9980	0.105*	
C8	0.2183 (5)	0.1208 (3)	1.0127 (3)	0.0856 (14)	
H8A	0.2512	0.1810	1.0188	0.103*	
H8B	0.2242	0.0975	1.0861	0.103*	
I1	0.18377 (3)	0.107898 (16)	0.365025 (19)	0.05948 (8)	
I2	0.65646 (3)	0.170576 (15)	0.988829 (18)	0.05505 (8)	
N1	0.3786 (3)	0.20013 (19)	0.7513 (2)	0.0624 (8)	
H1C	0.3307	0.2498	0.7324	0.075*	
H1D	0.4318	0.2087	0.8171	0.075*	
N2	0.3770 (3)	0.0320 (2)	0.6482 (2)	0.0651 (8)	
H2	0.3368	0.0405	0.5772	0.078*	
N3	0.1905 (3)	−0.07111 (17)	0.7482 (2)	0.0589 (8)	
H3C	0.2237	−0.0967	0.8128	0.071*	
H3D	0.0998	−0.0827	0.7316	0.071*	
N4	0.0290 (3)	0.1048 (2)	0.6231 (2)	0.0615 (8)	
H4C	0.0554	0.1257	0.5623	0.074*	
H4D	−0.0143	0.0531	0.6065	0.074*	
N5	0.0621 (3)	0.1560 (2)	0.8462 (2)	0.0604 (8)	
H5	0.0848	0.2141	0.8520	0.073*	
N6	0.3059 (3)	0.06852 (19)	0.9535 (2)	0.0606 (8)	
H6C	0.3036	0.0116	0.9731	0.073*	
H6D	0.3934	0.0874	0.9702	0.073*	

### Atomic displacement parameters (Å^2^)

**Table:**

	*U*^11^	*U*^22^	*U*^33^	*U*^12^	*U*^13^	*U*^23^
Cd1	0.03847 (13)	0.04408 (13)	0.04229 (12)	0.00397 (10)	0.01021 (10)	−0.00010 (9)
C1	0.070 (3)	0.110 (4)	0.072 (3)	−0.022 (3)	0.024 (2)	0.021 (2)
C2	0.048 (2)	0.121 (4)	0.074 (3)	0.001 (2)	0.025 (2)	−0.007 (3)
C3	0.075 (3)	0.082 (3)	0.108 (4)	0.028 (3)	0.022 (3)	−0.031 (3)
C4	0.101 (4)	0.066 (3)	0.081 (3)	0.015 (3)	0.003 (3)	−0.024 (2)
C5	0.057 (2)	0.077 (3)	0.098 (3)	0.023 (2)	0.001 (2)	0.009 (2)
C6	0.048 (2)	0.099 (3)	0.094 (3)	0.017 (2)	0.022 (2)	−0.014 (3)
C7	0.091 (4)	0.119 (4)	0.063 (2)	−0.002 (3)	0.039 (3)	−0.011 (2)
C8	0.117 (4)	0.097 (3)	0.044 (2)	−0.010 (3)	0.018 (2)	−0.015 (2)
I1	0.05661 (15)	0.05952 (15)	0.06234 (15)	−0.00248 (11)	0.01062 (12)	0.00141 (11)
I2	0.05468 (15)	0.05668 (14)	0.05193 (13)	−0.00187 (11)	0.00440 (11)	0.00483 (9)
N1	0.0556 (18)	0.0579 (17)	0.0728 (19)	−0.0036 (15)	0.0085 (16)	0.0135 (15)
N2	0.0564 (19)	0.087 (2)	0.0537 (16)	0.0093 (18)	0.0138 (15)	−0.0073 (16)
N3	0.067 (2)	0.0489 (16)	0.0553 (16)	−0.0018 (15)	−0.0044 (15)	−0.0007 (13)
N4	0.0578 (18)	0.074 (2)	0.0518 (16)	0.0084 (16)	0.0064 (15)	0.0092 (14)
N5	0.064 (2)	0.0563 (17)	0.0658 (18)	0.0051 (15)	0.0250 (17)	−0.0098 (14)
N6	0.074 (2)	0.0551 (17)	0.0481 (15)	−0.0121 (15)	−0.0012 (15)	0.0081 (13)

### Geometric parameters (Å, °)

**Table:**

Cd1—N5	2.352 (3)	C5—H5A	0.9700
Cd1—N1	2.357 (3)	C5—H5B	0.9700
Cd1—N2	2.365 (3)	C6—N5	1.463 (5)
Cd1—N3	2.366 (3)	C6—H6A	0.9700
Cd1—N6	2.380 (3)	C6—H6B	0.9700
Cd1—N4	2.381 (3)	C7—N5	1.464 (5)
C1—N1	1.457 (5)	C7—C8	1.478 (6)
C1—C2	1.490 (6)	C7—H7A	0.9700
C1—H1A	0.9700	C7—H7B	0.9700
C1—H1B	0.9700	C8—N6	1.465 (5)
C2—N2	1.455 (5)	C8—H8A	0.9700
C2—H2A	0.9700	C8—H8B	0.9700
C2—H2B	0.9700	I2—N1	3.685 (3)
C3—N2	1.478 (5)	N1—H1C	0.9000
C3—C4	1.497 (6)	N1—H1D	0.9000
C3—H3A	0.9700	N2—H2	0.9100
C3—H3B	0.9700	N3—H3C	0.9000
C4—N3	1.441 (5)	N3—H3D	0.9000
C4—H4A	0.9700	N4—H4C	0.9000
C4—H4B	0.9700	N4—H4D	0.9000
C5—N4	1.460 (5)	N5—H5	0.9100
C5—C6	1.478 (6)	N6—H6C	0.9000
C5—I1	4.854 (4)	N6—H6D	0.9000
			
N5—Cd1—N1	99.56 (11)	H6A—C6—H6B	108.1
N5—Cd1—N2	167.86 (11)	N5—C7—C8	110.1 (4)
N1—Cd1—N2	74.45 (11)	N5—C7—H7A	109.6
N5—Cd1—N3	113.37 (11)	C8—C7—H7A	109.6
N1—Cd1—N3	146.11 (11)	N5—C7—H7B	109.6
N2—Cd1—N3	74.54 (11)	C8—C7—H7B	109.6
N5—Cd1—N6	74.53 (10)	H7A—C7—H7B	108.2
N1—Cd1—N6	91.23 (10)	N6—C8—C7	111.5 (3)
N2—Cd1—N6	115.63 (11)	N6—C8—H8A	109.3
N3—Cd1—N6	90.05 (10)	C7—C8—H8A	109.3
N5—Cd1—N4	73.76 (10)	N6—C8—H8B	109.3
N1—Cd1—N4	107.61 (11)	C7—C8—H8B	109.3
N2—Cd1—N4	97.70 (11)	H8A—C8—H8B	108.0
N3—Cd1—N4	89.76 (10)	C1—N1—Cd1	110.3 (2)
N6—Cd1—N4	145.29 (10)	C1—N1—I2	94.7 (2)
N1—C1—C2	111.0 (3)	Cd1—N1—I2	104.93 (9)
N1—C1—H1A	109.4	C1—N1—H1C	109.6
C2—C1—H1A	109.4	Cd1—N1—H1C	109.6
N1—C1—H1B	109.4	I2—N1—H1C	126.4
C2—C1—H1B	109.4	C1—N1—H1D	109.6
H1A—C1—H1B	108.0	Cd1—N1—H1D	109.6
N2—C2—C1	110.6 (3)	H1C—N1—H1D	108.1
N2—C2—H2A	109.5	C2—N2—C3	116.1 (3)
C1—C2—H2A	109.5	C2—N2—Cd1	107.4 (2)
N2—C2—H2B	109.5	C3—N2—Cd1	107.2 (2)
C1—C2—H2B	109.5	C2—N2—H2	108.6
H2A—C2—H2B	108.1	C3—N2—H2	108.6
N2—C3—C4	109.9 (4)	Cd1—N2—H2	108.6
N2—C3—H3A	109.7	C4—N3—Cd1	110.0 (2)
C4—C3—H3A	109.7	C4—N3—H3C	109.7
N2—C3—H3B	109.7	Cd1—N3—H3C	109.7
C4—C3—H3B	109.7	C4—N3—H3D	109.7
H3A—C3—H3B	108.2	Cd1—N3—H3D	109.7
N3—C4—C3	110.6 (3)	H3C—N3—H3D	108.2
N3—C4—H4A	109.5	C5—N4—Cd1	109.7 (2)
C3—C4—H4A	109.5	C5—N4—H4C	109.7
N3—C4—H4B	109.5	Cd1—N4—H4C	109.7
C3—C4—H4B	109.5	C5—N4—H4D	109.7
H4A—C4—H4B	108.1	Cd1—N4—H4D	109.7
N4—C5—C6	109.5 (3)	H4C—N4—H4D	108.2
C6—C5—I1	145.2 (2)	C6—N5—C7	115.7 (3)
N4—C5—H5A	109.8	C6—N5—Cd1	108.9 (2)
C6—C5—H5A	109.8	C7—N5—Cd1	107.1 (3)
I1—C5—H5A	95.1	C6—N5—H5	108.3
N4—C5—H5B	109.8	C7—N5—H5	108.3
C6—C5—H5B	109.8	Cd1—N5—H5	108.3
I1—C5—H5B	83.7	C8—N6—Cd1	109.2 (2)
H5A—C5—H5B	108.2	C8—N6—H6C	109.8
N5—C6—C5	110.7 (3)	Cd1—N6—H6C	109.8
N5—C6—H6A	109.5	C8—N6—H6D	109.8
C5—C6—H6A	109.5	Cd1—N6—H6D	109.8
N5—C6—H6B	109.5	H6C—N6—H6D	108.3
C5—C6—H6B	109.5		
			
N1—C1—C2—N2	58.9 (5)	N5—Cd1—N3—C4	159.6 (3)
N2—C3—C4—N3	−60.7 (5)	N1—Cd1—N3—C4	−35.0 (3)
N4—C5—C6—N5	60.3 (5)	N2—Cd1—N3—C4	−10.6 (3)
I1—C5—C6—N5	47.9 (7)	N6—Cd1—N3—C4	−127.3 (3)
N5—C7—C8—N6	−59.8 (5)	N4—Cd1—N3—C4	87.5 (3)
C2—C1—N1—Cd1	−36.4 (4)	C6—C5—N4—Cd1	−41.6 (4)
C2—C1—N1—I2	71.5 (3)	I1—C5—N4—Cd1	126.6 (3)
N5—Cd1—N1—C1	−161.1 (3)	N5—Cd1—N4—C5	13.1 (2)
N2—Cd1—N1—C1	8.0 (3)	N1—Cd1—N4—C5	−82.0 (3)
N3—Cd1—N1—C1	32.4 (3)	N2—Cd1—N4—C5	−158.1 (3)
N6—Cd1—N1—C1	124.3 (3)	N3—Cd1—N4—C5	127.6 (3)
N4—Cd1—N1—C1	−85.3 (3)	N6—Cd1—N4—C5	37.8 (3)
N5—Cd1—N1—I2	97.95 (10)	C5—C6—N5—C7	−166.7 (4)
N2—Cd1—N1—I2	−92.87 (11)	C5—C6—N5—Cd1	−46.1 (4)
N3—Cd1—N1—I2	−68.49 (19)	C8—C7—N5—C6	172.1 (4)
N6—Cd1—N1—I2	23.43 (10)	C8—C7—N5—Cd1	50.5 (4)
N4—Cd1—N1—I2	173.83 (9)	N1—Cd1—N5—C6	122.7 (3)
C1—C2—N2—C3	−168.3 (3)	N2—Cd1—N5—C6	63.4 (6)
C1—C2—N2—Cd1	−48.4 (4)	N3—Cd1—N5—C6	−65.5 (3)
C4—C3—N2—C2	167.6 (3)	N6—Cd1—N5—C6	−148.7 (3)
C4—C3—N2—Cd1	47.6 (4)	N4—Cd1—N5—C6	17.0 (3)
N5—Cd1—N2—C2	82.9 (6)	N1—Cd1—N5—C7	−111.5 (3)
N1—Cd1—N2—C2	21.2 (3)	N2—Cd1—N5—C7	−170.9 (5)
N3—Cd1—N2—C2	−145.0 (3)	N3—Cd1—N5—C7	60.3 (3)
N6—Cd1—N2—C2	−62.6 (3)	N6—Cd1—N5—C7	−22.9 (3)
N4—Cd1—N2—C2	127.4 (3)	N4—Cd1—N5—C7	142.8 (3)
N5—Cd1—N2—C3	−151.7 (5)	C7—C8—N6—Cd1	35.5 (4)
N1—Cd1—N2—C3	146.6 (3)	N5—Cd1—N6—C8	−6.3 (3)
N3—Cd1—N2—C3	−19.6 (3)	N1—Cd1—N6—C8	93.3 (3)
N6—Cd1—N2—C3	62.9 (3)	N2—Cd1—N6—C8	166.6 (3)
N4—Cd1—N2—C3	−107.2 (3)	N3—Cd1—N6—C8	−120.6 (3)
C3—C4—N3—Cd1	39.8 (4)	N4—Cd1—N6—C8	−30.9 (3)

### Hydrogen-bond geometry (Å, °)

**Table:**

*D*—H···*A*	*D*—H	H···*A*	*D*···*A*	*D*—H···*A*
N3—H3C···I2^i^	0.90	2.77	3.673 (3)	176
N6—H6C···I2^i^	0.90	2.82	3.709 (3)	168
N3—H3D···I1^ii^	0.90	2.87	3.759 (3)	168
N4—H4D···I1^ii^	0.90	3.02	3.873 (3)	159
N5—H5···I1^iii^	0.91	2.87	3.778 (3)	174
N1—H1D···I2	0.90	2.85	3.685 (3)	155
N2—H2···I1	0.91	2.98	3.869 (3)	167
N4—H4C···I1	0.90	2.96	3.789 (3)	153
N6—H6D···I2	0.90	2.86	3.751 (3)	169

Symmetry codes: (i) −*x*+1, −*y*, −*z*+2; (ii) −*x*, −*y*, −*z*+1; (iii) *x*, −*y*+1/2, *z*+1/2.
